# CV‐SCAN (Crystal Violet Staining for Colitis‐Associated Neoplasia): A Novel Endoscopic Staining Method to Detect Paneth Cell Metaplasia and Ulcerative Colitis (UC)‐Associated Neoplasia in UC

**DOI:** 10.1111/den.70096

**Published:** 2026-01-07

**Authors:** Akira Tomioka, Nanoka Chiya, Chie Kurihara, Yoshikiyo Okada, Kazuyuki Narimatsu, Masaaki Higashiyama, Shunsuke Komoto, Ryota Hokari

**Affiliations:** ^1^ National Defense Medical College Hospital Tokorozawa‐Shi Saitama‐Ken Japan

**Keywords:** colorectal neoplasms, gastrointestinal endoscopy, metaplasia, staining and labeling, ulcerative colitis

## Abstract

**Objectives:**

Paneth cell metaplasia (PCM), a metaplastic change associated with chronic inflammation in ulcerative colitis (UC), may be linked to UC‐associated neoplasia (UCAN). However, no endoscopic method currently exists for detecting PCM. This study aimed to develop and validate a novel endoscopic staining technique—CV‐SCAN—for identifying PCM and UCAN, and to explore the molecular characteristics of the stained areas.

**Methods:**

This retrospective observational study included 131 patients with UC undergoing surveillance colonoscopy. CV‐SCAN involved spraying an ultra‐diluted solution (0.006%) of crystal violet from the descending colon to the rectum. Biopsies were obtained from stained and non‐stained areas and evaluated histologically and molecularly. RNA expression profiles were analyzed via microarray and real‐time RT‐PCR. The diagnostic performance of CV‐SCAN for detecting PCM was assessed, along with its correlation with UCAN history.

**Results:**

CV‐SCAN visualized sharply demarcated, purple‐stained areas corresponding to PCM or UCAN. PCM was significantly associated with a history of UCAN. Uniform, dark staining was characteristic of PCM, while UCAN showed heterogeneous staining with small round pits. CV‐SCAN achieved a sensitivity of 81.3% and a specificity of 84.9% for PCM detection. Molecular analysis revealed upregulation of Paneth cell–specific (DEFA5, DEFA6), small intestinal (CCL25, APOC3), and UCAN‐associated (IL17RC) genes, along with downregulation of SATB2 in stained areas.

**Conclusions:**

CV‐SCAN is a novel and effective endoscopic staining method for detecting PCM and UCAN in patients with UC. It enables risk stratification through direct visualization of precancerous changes and may facilitate early detection and targeted surveillance.

## 1 Introduction

Ulcerative colitis (UC) is a chronic inflammatory bowel disease characterized by diffuse colonic mucosal inflammation. In Japan, the prevalence of UC continues to rise, with an estimated 300,000 individuals currently affected [1, 2].

A major complication of long‐standing UC is the development of UC‐associated neoplasia (UCAN), which encompasses low‐grade dysplasia (LGD), high‐grade dysplasia, and UC‐associated colorectal cancer (UC‐CRC). UCAN arises through a molecularly distinct dysplasia–carcinoma pathway characterized by early p53 alterations [3, 4]. Given these characteristics, UCAN often presents as multifocal lesions within a precancerous mucosal background, and total colectomy may be considered over local resection in selected cases [5].

Despite advances in chromoendoscopy and image‐enhanced endoscopy, UCAN remains difficult to detect. Lesions are frequently flat with indistinct borders on heterogeneous, chronically inflamed mucosa, and chronic inflammation can distort pit patterns [6, 7, 8]. Therefore, additional markers that visualize the “field” of cancer risk are needed to improve surveillance strategies.

Metaplastic changes arising from chronic inflammation have recently gained attention as potential precancerous markers, such as gastric intestinal metaplasia used to stratify cancer risk [9, 10].

Among these, Paneth cell metaplasia (PCM)—defined as the ectopic appearance of Paneth cells in the distal colon—is a recognized histological finding in UC with chronic inflammation and has been linked to UCAN [11, 12]. SATB2 dysregulation has also been implicated in the development of PCM and small intestinal metaplasia, suggesting that PCM may represent a precancerous metaplastic phenotype in UC [13, 14, 15].

**FIGURE 1:**
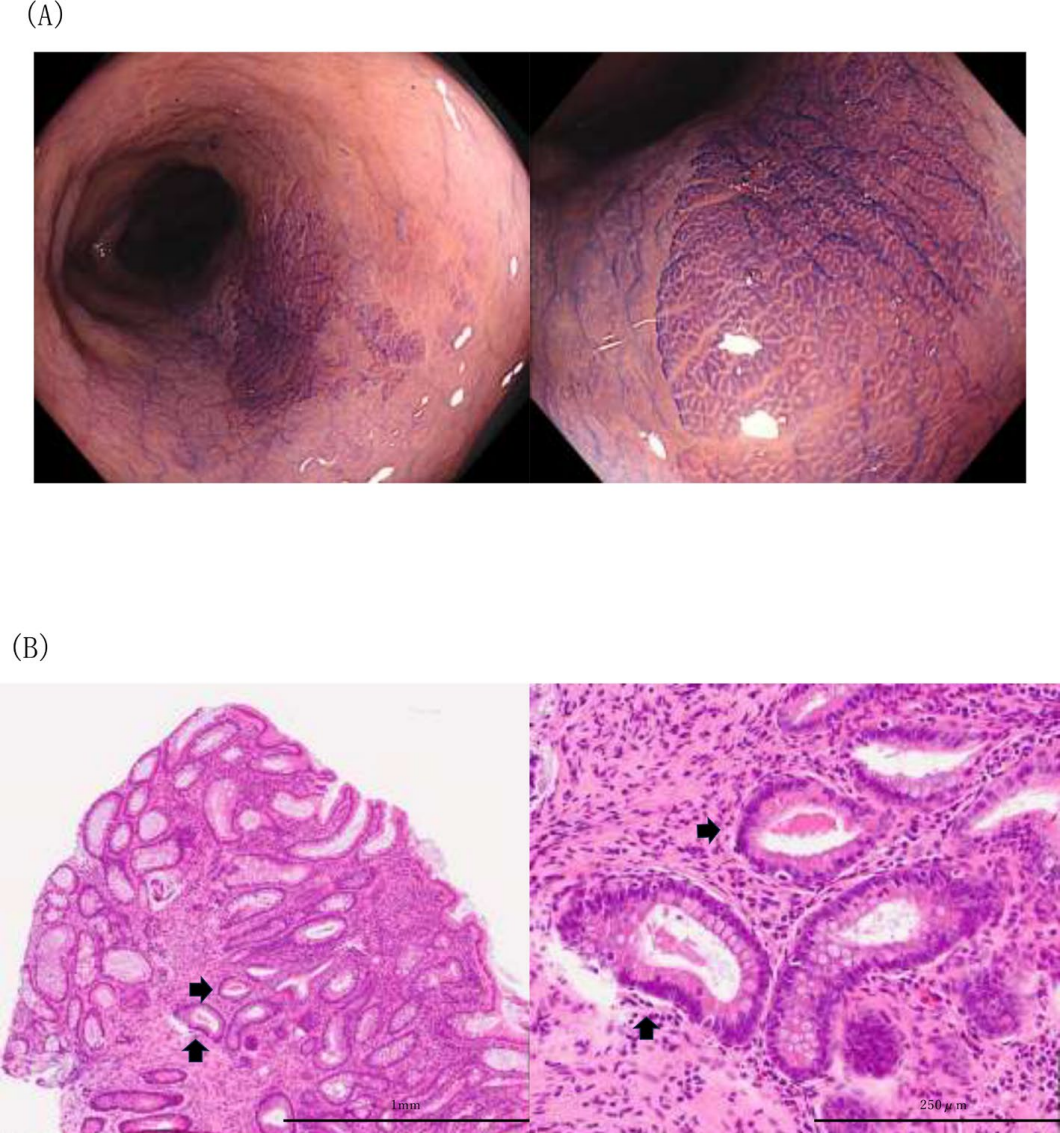
Endoscopic and histopathological images of crystal violet‐stainable areas in ulcerative colitis (UC). (A) Endoscopic image. (B) Histopathological image (hematoxylin and eosin staining). Crystal violet staining revealed variations in staining intensity within the colonic mucosa of patients with UC, with clearly demarcated areas exhibiting positive staining (A). Corresponding biopsy specimens from these sites demonstrated numerous characteristic eosinophilic intracellular granules within areas of Paneth cell metaplasia (PCM) (arrows) (B).

Despite its potential clinical relevance, PCM is currently diagnosed only by histopathological evaluation of biopsy specimens, as no method for endoscopic visualization has been established. In this study, we developed and applied a novel endoscopic staining technique, CV‐SCAN, using diluted crystal violet (CV) to assess the feasibility of endoscopically detecting PCM (Figure 1). The concept arose from an incidental observation during pit pattern assessment: in some patients with UC, diluted CV dye was retained more intensively in certain mucosal areas despite a normal appearance on white light imaging.

In this study, we applied CV‐SCAN in patients with UC undergoing surveillance colonoscopy and evaluated the following:

1. the diagnostic accuracy of CV‐SCAN for detecting PCM,
2. its association with UCAN and UCAN history, and
3. the molecular characteristics of CV‐SCAN–positive areas using gene expression profiling.

## 2 Methods

### 2.1 Study Design and Ethical Considerations

This retrospective observational study was conducted at the National Defense Medical College Hospital between March 2023 and April 2024. Ultra‐diluted CV (0.006%) was sprayed from the descending colon to the rectum during routine surveillance colonoscopy in patients with UC, after written informed consent for the staining procedure and biopsies. No additional intervention beyond standard clinical care was performed. Clinical, endoscopic, and histopathological data were collected from medical records. The study protocol was approved by the institutional ethics committee (approval no.: 4957).

### 2.2 Patients

**FIGURE 2:**
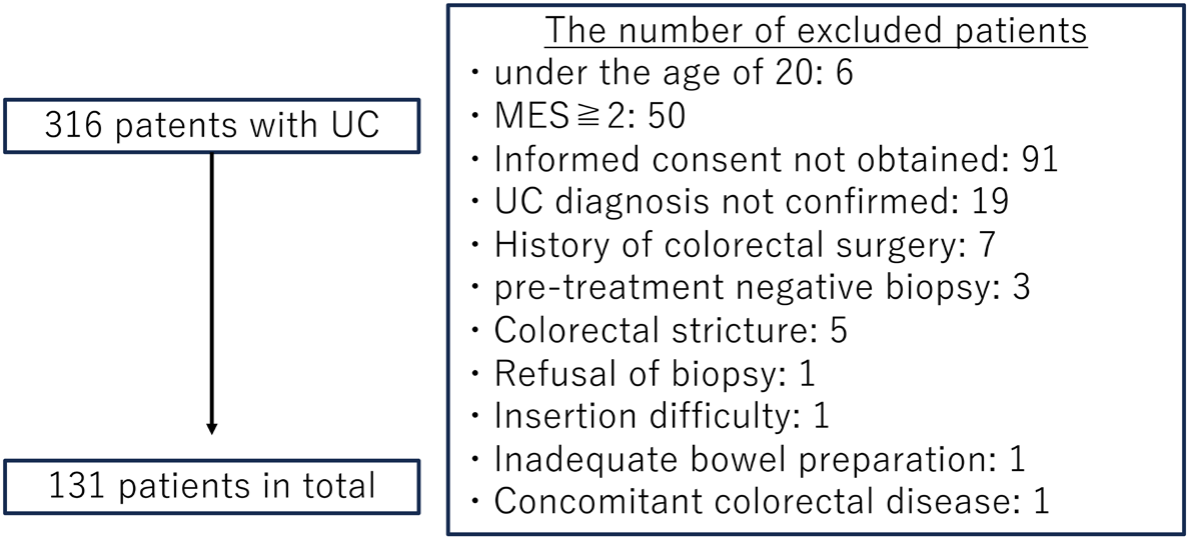
Eligible patients and exclusion criteria. This study included all patients diagnosed with ulcerative colitis (UC) who underwent colonoscopy at National Defense Medical College Hospital between March 2023 and April 2024. Eligible participants were patients aged 20 years or older with a confirmed diagnosis of UC who underwent colonoscopy during the study period. Exclusion criteria included the following: Age < 20 years, active disease with a Mayo endoscopic score ≥ 2, lack of consent for crystal violet staining or biopsy, non‐UC diagnosis, history of colorectal resection, biopsy performed for pre‐treatment evaluation, presence of intestinal strictures, difficulty with scope insertion, inadequate bowel preparation, or comorbid colorectal diseases. A total of 131 patients were included in the analysis.

Eligible participants were patients aged 20 years or older with a confirmed diagnosis of UC who underwent colonoscopy during the study period. Exclusion criteria included: age < 20 years, active disease with a Mayo endoscopic score ≥ 2, lack of consent for crystal violet staining or biopsy, non‐UC diagnosis, history of colorectal resection, biopsy performed for pre‐treatment evaluation, presence of intestinal strictures, difficulty with scope insertion, inadequate bowel preparation, or comorbid colorectal diseases. A total of 131 patients were included in the analysis (Figure 2).

### 2.3 Endoscopic Procedure and Staining Evaluation

**FIGURE 3:**
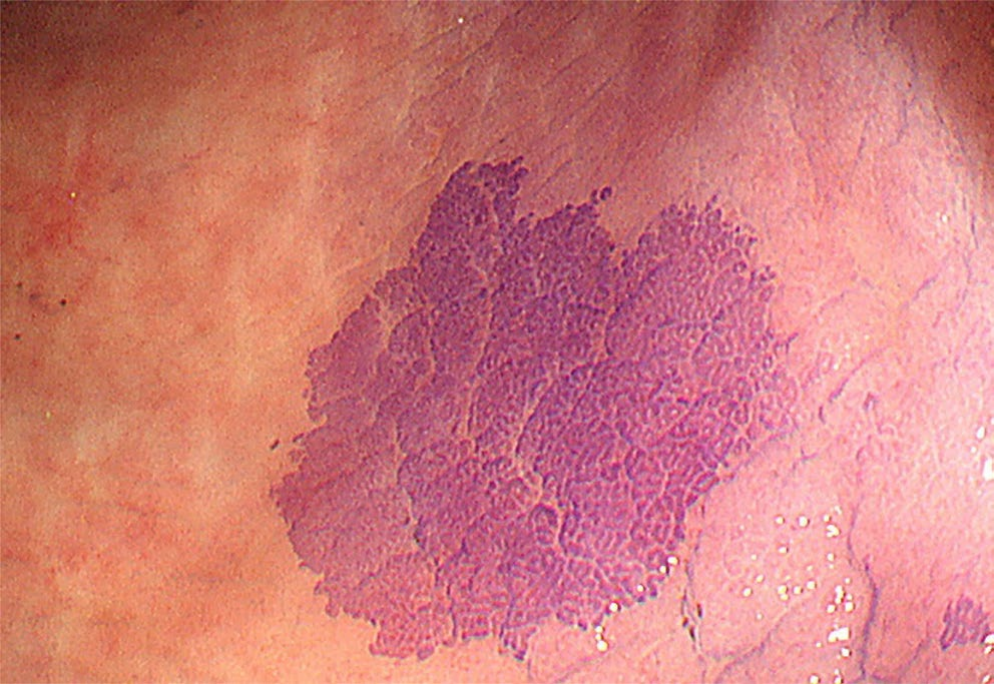
Endoscopic image showing “darkly stained area” with CV‐SCAN (crystal violet staining for colitis‐associated neoplasia). Intense staining was defined as distinct purple coloration with clearly demarcated borders from the surrounding mucosa. In contrast, faint mucosal staining resulting from prolonged exposure to crystal violet due to gravity or other factors was not classified as intense staining.

**FIGURE 4:**
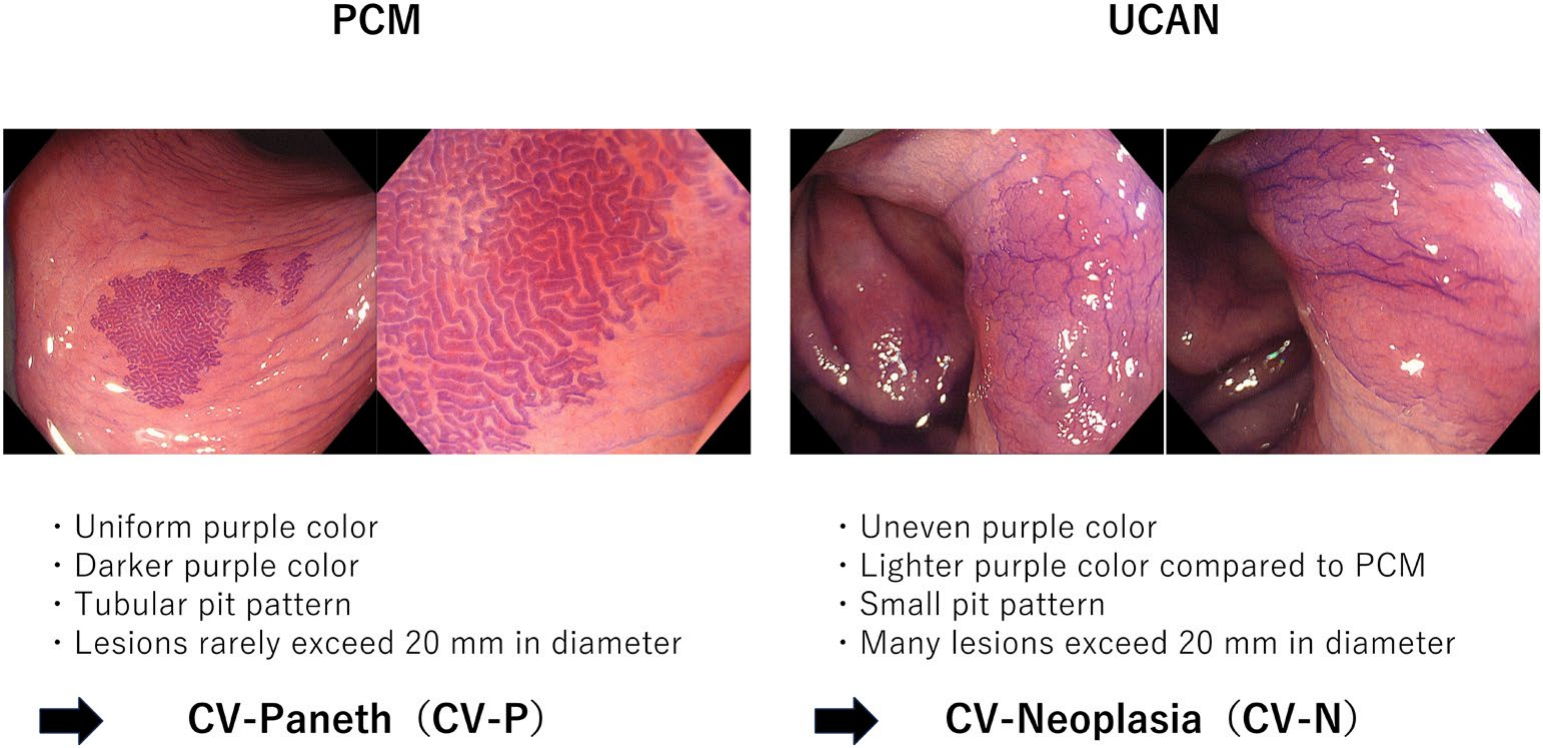
Endoscopic classification of intense crystal violet staining. Both Paneth cell metaplasia (PCM) and ulcerative colitis‐associated neoplasia (UCAN) may appear as intensely stained areas with crystal violet staining. However, their staining characteristics differ. UCAN typically presents as unevenly stained purple areas, whereas PCM shows more uniform staining. For clarity, crystal violet‐positive PCM is referred to as crystal violet–Paneth cell metaplasia (CV‐P), and crystal violet‐positive UCAN as crystal violet–neoplasia (CV‐N).

The degree of mucosal inflammation was assessed during colonoscope insertion. After reaching the cecum, standard observation was performed from the cecum to the transverse colon, followed by CV staining from the descending colon to the rectum. A diluted 0.006% CV solution was sprayed gently and evenly via a catheter (Olympus, Tokyo, Japan) to minimize background staining. Use of the spray catheter allowed homogenous distribution of the minimal effective volume; approximately 40 mL of 0.006% CV was applied per procedure. Areas that demonstrated deep purple staining with clear demarcation from the surrounding mucosa were defined as “darkly stained areas” (Figure 3). Faint or diffuse mucosal staining due to prolonged dye contact from gravity was not considered dark staining. Furthermore, staining patterns were subclassified into CV‐P (crystal violet–Paneth cell metaplasia: uniform purple staining with tubular pits resembling Type III_L_ pit pattern) and CV‐N (crystal violet–neoplasia: heterogeneous purple staining with densely distributed small pits that appeared smaller than normal Type I pit pattern) (Figure 4). CV‐N was hypothesized to represent neoplastic lesions, and additional biopsies or therapeutic interventions were considered for these areas.

When darkly stained areas were observed, targeted biopsies were obtained from both stained and adjacent non‐stained mucosa. In the absence of staining, biopsies were performed from at least one site each in the sigmoid colon and rectum.

### 2.4 Histopathological and Molecular Evaluation

Histological assessment of PCM was performed using hematoxylin and eosin (HE) staining. Paneth cell counts were evaluated in five high‐power fields (5 HPFs) at 400× magnification. Specimens lacking the glandular crypt base were excluded.

For suspected UCAN, additional Ki‐67 and p53 immunohistochemical staining was performed, and diagnoses were based on characteristic histological features, including bottom‐up morphogenesis [16, 17].

Gene expression profiling was first performed using microarray on biopsy samples from normal colonic mucosa, CV‐P–stained areas, and adjacent non‐stained areas, and selected genes were then validated by quantitative reverse transcription–PCR (qRT‐PCR) in 14 patients with UC using paired CV‐P–stained and non‐stained mucosa. Relative expression levels were calculated using the ΔΔCt method and normalized to β‐glucuronidase.

### 2.5 Statistical Analysis

Associations between dark staining and UCAN, as well as between PCM and UCAN, were assessed using Fisher's exact test. Receiver operating characteristic (ROC) curve analysis was conducted to determine the optimal Paneth cell count cutoff for identifying stained areas. Comparisons between the stained group and non‐stained group were first performed using univariate analyses: the Mann–Whitney *U* test for continuous variables and the chi‐squared test or Fisher's exact test for categorical variables. Univariate odds ratios (ORs) with 95% confidence intervals (CIs) were calculated for categorical variables. Variables with *p* < 0.10 were entered into a multivariate logistic regression model to identify independent factors associated with CV‐SCAN positivity. Similarly, comparisons between the PCM‐positive group and PCM‐negative group were conducted using the same univariate approach, followed by multivariate logistic regression for variables with *p* < 0.10 to determine independent predictors of PCM.

All statistical tests were two‐sided, and *p* < 0.05 was considered statistically significant. Analyses were performed using JMP Pro 16 (SAS Institute, Cary, NC, USA).

## 3 Results

### 3.1 Detection Rate and Accuracy of PCM by CV‐SCAN

Patient characteristics are summarized in Table 1. A total of 568 biopsy specimens were collected from the descending colon to the rectum. CV‐SCAN enabled clear visualization of PCM without the need for special bowel preparation or mucolytic cleaning. Staining occurred immediately after dye application. The stained areas ranged from tiny lesions (as small as a single pit) to larger areas exceeding 20 mm in diameter, and their number varied among patients.

**TABLE 1:** Patient characteristics.

Sex	67 males (51%), 64 females (49%)
Age	21–84 years old (median, 52 years old)
Disease extent	Pancolitis: 98 (75%), left‐sided colitis: 27 (21%), proctitis: 6 (5%)
Disease duration	0.5–46 years (median, 13 years)
Intensely stained group	60 patients (46%)
History of UCAN	10 patients (8%)

*Note:* The clinical characteristics of the enrolled patients are summarized in this table. 10 patients (8%) had a history of ulcerative colitis‐associated neoplasia (UCAN).

**FIGURE 5:**
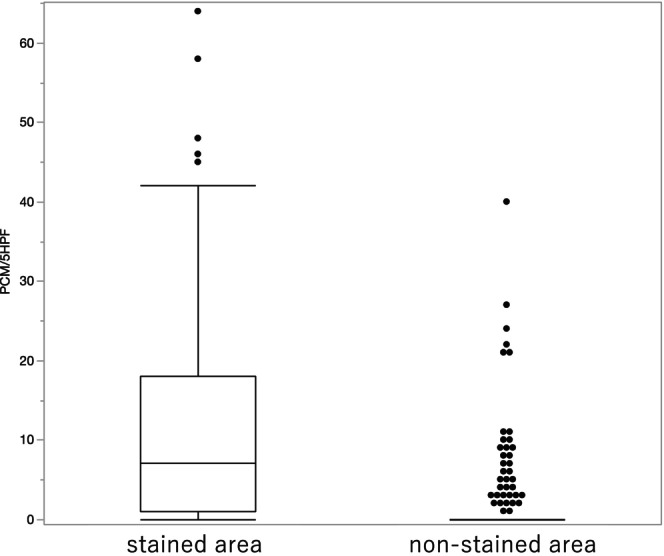
Distribution of Paneth cell counts in biopsy specimens. Paneth cell counts were evaluated in five high‐power fields (5 HPFs) of biopsy specimens obtained from stained and non‐stained areas by CV‐SCAN (crystal violet staining for colitis‐associated neoplasia). In stained areas, the minimum, median, and maximum Paneth cell counts were 0, 7, and 64, respectively. In non‐stained areas, the corresponding values were 0, 0, and 40.

CV‐SCAN identified PCM with a sensitivity of 81.3% and a specificity of 84.9% (Table 2). The median number of Paneth cells per five high‐power fields (5 HPFs) was 7 in darkly stained areas and 0 in stained areas (Figure 5).

**TABLE 2:** Sensitivity and specificity of Paneth cell metaplasia (PCM) detection by CV‐SCAN (crystal violet staining for colitis‐associated neoplasia).

	Pathological diagnosis		Total
	PCM present	PCM absent	
Darkly stained	187	51	238
Non‐stained	43	287	330
Total	230	338	568

*Note:* A total of 568 biopsy specimens were obtained from the left colon. CV‐SCAN demonstrated a sensitivity of 81.3% and a specificity of 84.9% for the detection of PCM.

**FIGURE 6:**
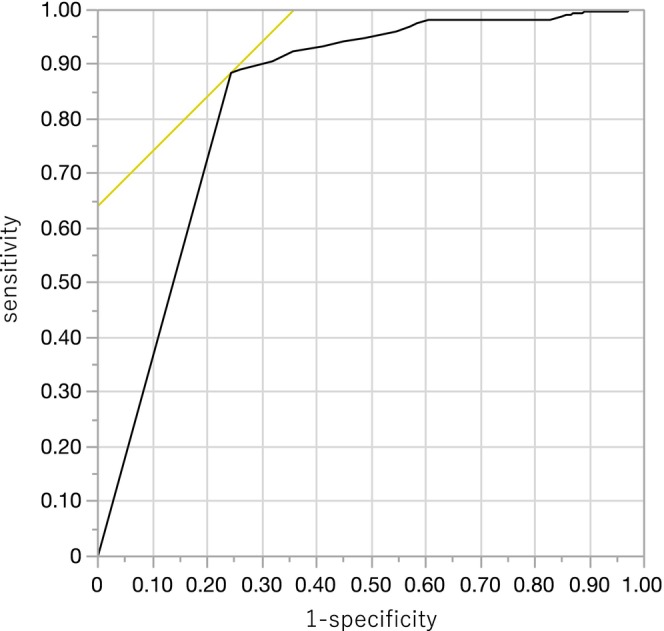
Receiver operating characteristic (ROC) curve for Paneth cell count in predicting crystal violet staining. An ROC curve was constructed to determine the optimal cutoff value for predicting the presence or absence of crystal violet staining based on Paneth cell counts. The area under the curve (AUC) was 0.83549, forming a convex shape in the upper left quadrant, indicating good discriminative ability. A cutoff value of 0 was identified as optimal. Using this threshold, sensitivity was 88.5% and specificity was 76.4%.

ROC curve analysis yielded an area under the curve (AUC) of 0.835. A Paneth cell count of 0 was determined to be the optimal cutoff, resulting in a sensitivity of 88.5% and a specificity of 76.4% (Figure 6).

**FIGURE 7:**
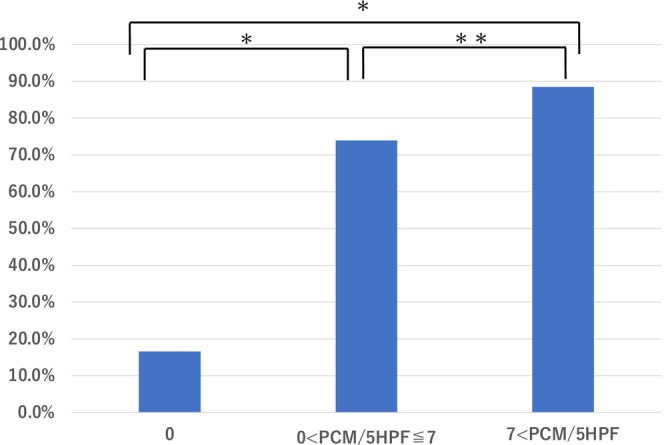
Comparison of crystal violet staining intensity by Paneth cell count. Biopsy specimens obtained from intensely stained areas were stratified into three groups based on Paneth cell counts per 5 high‐power fields (HPF): 0, > 0–≤ 7, and > 7 cells. The median Paneth cell count in intensely stained areas was 7/5 HPF. The vertical axis of the bar graph represents the proportion of specimens showing intense staining: 16.6% in the group with 0 Paneth cells, 73.9% in the > 0–≤ 7 group, and 88.5% in the > 7 group. Chi‐square tests were conducted to evaluate the association between Paneth cell count and the presence of intense staining. Statistically significant associations were found between each pair of groups (*p < 0.0001, **p < 0.0057).

Biopsy specimens from stained areas were further categorized into three groups based on Paneth cell count: 0, 1–7, and > 7 cells/5 HPFs. A statistically significant association was observed between the number of Paneth cells and the presence of dark staining (Figure 7).

Stained areas occurred on a variety of endoscopic backgrounds, including mucosa that appeared normal, erythematous, or whitish and scarred under white light endoscopy. In many cases, CV‐SCAN–positive mucosa could not be distinguished from the surrounding tissue before dye application, indicating that the technique highlights changes not appreciable on routine inspection.

### 3.2 Association Between PCM or UCAN History and CV‐SCAN Staining

Table 3 shows the comparison between the stained and non‐stained groups. In univariate analysis, male sex (OR 2.20, 95% CI 1.09–4.44, *p* = 0.035), longer disease duration (*p* = 0.027), and a history of immunomodulator use (OR 2.73, 95% CI 1.34–5.58, *p* = 0.008) were significantly associated with CV‐SCAN positivity. In multivariate analysis, male sex (adjusted OR 2.18, 95% CI 1.00–4.87, *p* = 0.049) and longer disease duration (adjusted OR 1.05 per year, 95% CI 1.00–1.10, *p* = 0.041) remained independent predictors, whereas a history of tacrolimus use showed an inverse association (adjusted OR 0.24, 95% CI 0.08–0.77, *p* = 0.017).

**TABLE 3:** Characteristics of two patient groups: Crystal violet staining intensity and presence of Paneth cell metaplasia (PCM).

Variable	Stained group (*n* = 60)	Non‐stained group (*n* = 71)	Univariate OR (95% CI)	*p*	Multivariate OR (95% CI)	*p*
Sex (male/female)	37 (61.7%)/23 (38.3%)	30 (42%)/41 (58%)	2.20 (1.09–4.44)	0.035	2.18 (1.00–4.87)	0.049
Age (min–median–max)	22–55–82 years	21–51–84 years	—	0.354	1.00 (0.98–1.03)	0.835
Disease extent	Pancolitis: 46 (77%), left‐sided colitis: 14 (23%), proctitis: 0 (0%)	Pancolitis: 52 (73%), left‐sided colitis: 13 (18%), proctitis: 6 (8%)	—	0.063	1.15 (0.44–3.03)	0.773
Disease duration (min–median–max)	2–14–46 years	0.5–12–40 years	—	0.027	1.05 (1.00–1.10)	0.041
History of steroid use (yes/no)	44 (73%)/16 (27%)	42 (59%)/29 (41%)	1.90 (0.90–3.99)	0.1	1.61 (0.60–4.31)	0.347
History of immunomodulator use (yes/no)	40 (67%)/20 (33%)	30 (42%)/41 (58%)	2.73 (1.34–5.58)	0.008	2.26 (0.88–5.83)	0.091
History of biologics use (yes/no)	19 (32%)/41 (68%)	17 (24%)/54 (76%)	1.47 (0.68–3.18)	0.335	1.92 (0.64–5.78)	0.245
History of Janus kinase (JAK)inhibitor use (yes/no)	12 (20%)/48 (80%)	9 (13%)/62 (87%)	1.72 (0.62–4.42)	0.34	3.02 (0.97–9.40)	0.056
History of tacrolimus use (yes/no)	9 (15%)/51 (85%)	19 (27%)/52 (73%)	2.07 (0.86–5.00)	0.135	0.24 (0.08–0.77)	0.017

Variable	PCM‐positive group (*n* = 65)	PCM‐negative group (*n* = 66)	Univariate OR (95% CI)	*p*	Multivariate OR (95% CI)	*p*
Sex (male/female)	35 (54%)/30 (46%)	32 (48%)/44 (52%)	1.17 (0.58–2.36)	0.702	1.14 (0.40–3.25)	0.808
Age (min–median–max)	22–55–82 years	21–51.5–84 years	—	0.286	1.00 (0.97–1.03)	0.946
Disease extent	Pancolitis: 50 (77%), left‐sided colitis: 14 (22%), proctitis: 1 (2%)	Pancolitis: 48 (73%), left‐sided colitis: 13 (20%), proctitis: 5 (8%)	—	0.939	1.09 (0.44–2.70)	0.859
Disease duration (min–median–max)	2–16–46 years	0.5–10–40 years	—	0.001	1.09 (1.04–1.15)	0.001
History of steroid use (yes/no)	45 (69%)/20 (31%)	41 (62%)/25 (38%)	1.24 (0.59–2.64)	0.702	1.14 (0.40–3.25)	0.808
History of immunomodulator use (yes/no)	41 (63%)/24 (37%)	29 (44%)/37 (56%)	1.97 (0.96–4.03)	0.073	2.26 (0.88–5.83)	0.091
History of biologics use (yes/no)	20 (31%)/45 (69%)	16 (24%)/50 (76%)	1.28 (0.59–2.78)	0.56	1.92 (0.64–5.78)	0.245
History of Janus kinase (JAK)inhibitor use (yes/no)	16 (25%)/49 (75%)	5 (8%)/61 (92%)	3.73 (1.27–10.95)	0.016	7.86 (2.24–27.58)	0.001
History of tacrolimus use (yes/no)	12 (18%)/53 (82%)	16 (24%)/50 (76%)	0.65 (0.28–1.52)	0.392	0.47 (0.15–1.47)	0.192

*Note:* This table presents the clinical details of two sets of groups. The first comparison is between the intensely stained group, defined as patients with at least one area of intense crystal violet staining, and the non‐intensely stained group, defined as those without any intensely stained areas. The second comparison is between the PCM‐positive group, consisting of patients with at least one biopsy showing PCM, and the PCM‐negative group, with no PCM detected on biopsy. Values are presented as numbers (%) or minimum–median–maximum. Univariate odds ratios (ORs) with 95% confidence intervals (CIs) were calculated for categorical variables. Variables with *p* < 0.10 in univariate analysis were included in the multivariate logistic regression model. Adjusted ORs with 95% CIs are shown. In this analysis, patients with proctitis were excluded from the evaluation of disease extent.

When patients were stratified by PCM status, disease duration was significantly longer in the PCM‐positive group, and a history of JAK inhibitor use was strongly associated with PCM (adjusted OR 7.86, 95% CI 2.24–27.58, *p* = 0.001).

Table 4 summarizes the relationship between PCM, CV‐SCAN positivity, and UCAN history. Both the presence of PCM and CV‐SCAN positivity were significantly associated with a history of UCAN. All patients with a history of UCAN showed positive CV‐SCAN staining in the left colon. Because CV‐SCAN was applied only from the descending colon to the rectum, the diagnostic performance of the technique in the right colon could not be assessed; no right‐sided UCAN lesions were detected during the study period.

**TABLE 4:** Association between ulcerative colitis‐associated neoplasia (UCAN), Paneth cell metaplasia (PCM), and intense crystal violet staining.

		History of UCAN		Total
		Yes	No	
PCM	Positive	10	55	65
	Negative	0	66	66
Total		10	121	131

		History of UCAN		Total
		Yes	No	
CV‐SCAN	Positive	10	50	60
	Negative	0	71	71
Total		10	121	131

*Note:* A significant association was observed between the presence of PCM in biopsy specimens and the occurrence of UCAN (*p* = 0.0013). Similarly, the intensely stained group, defined by the presence of at least one intensely crystal violet‐stained area, also showed a significant association with UCAN (*p* = 0.00024).

### 3.3 Detection Rate and Accuracy of UCAN by CV‐N Pattern

**FIGURE 8:**
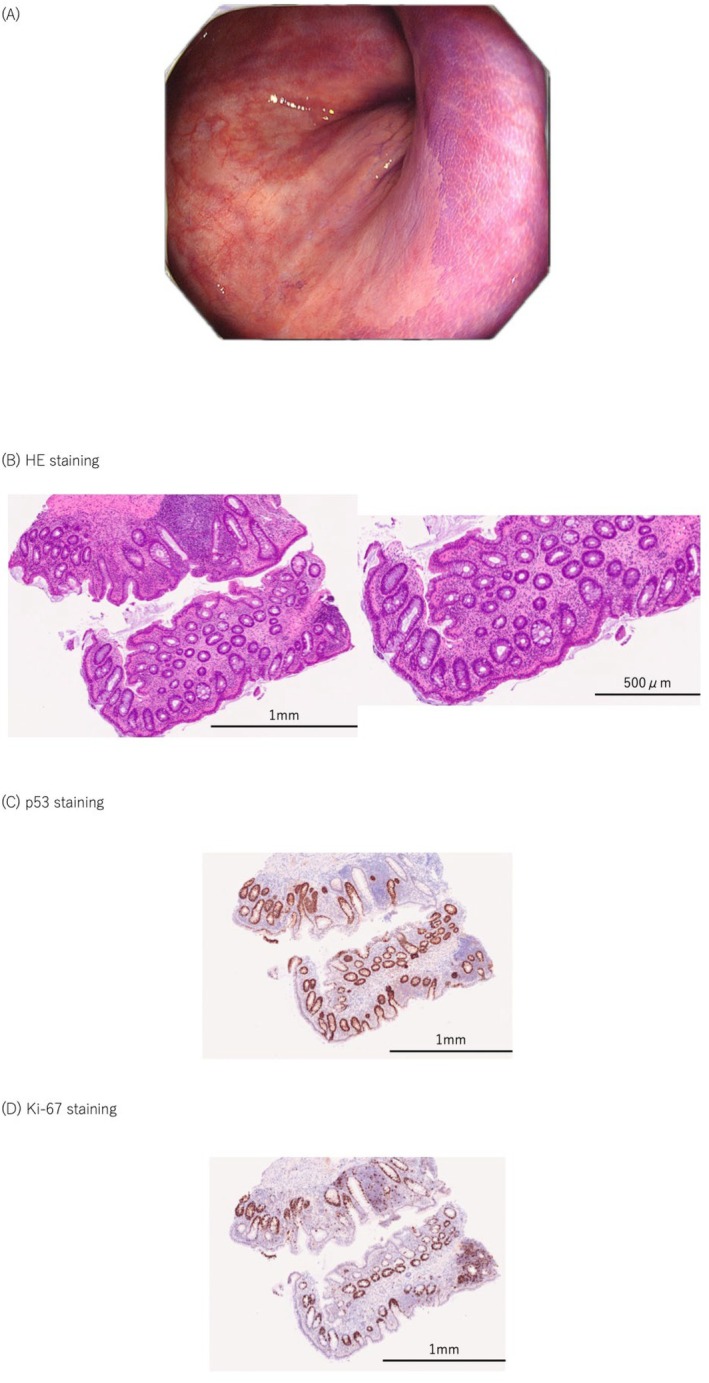
Representative endoscopic and histopathological features of crystal violet‐neoplasia (CV‐N)–positive low‐grade dysplasia (LDG) in ulcerative colitis. (A) Endoscopic image of a representative CV‐N–positive lesion. (B) Histopathological images showing low‐grade dysplasia on hematoxylin and eosin (HE) staining. (C) Immunohistochemical staining for p53. (D) Immunohistochemical staining for Ki‐67. This lesion demonstrated a clear CV‐N–positive staining pattern on crystal violet staining for colitis‐associated neoplasia (CV‐SCAN). Histopathological evaluation revealed LDG with characteristic architectural and cytological abnormalities on HE staining. Immunohistochemistry showed p53 overexpression and a Ki‐67 labeling pattern consistent with bottom‐up morphogenesis, supporting dysplastic transformation arising from the basal crypt epithelium. Taken together, these findings confirmed the diagnosis of LDG.

In this study, CV‐SCAN staining revealed eight lesions with the CV‐N pattern in three patients. A total of 31 biopsy specimens were obtained from both CV‐N–positive areas and adjacent regions to evaluate the detection rate and diagnostic accuracy of UCAN using CV‐SCAN. All eight CV‐N–positive lesions were histologically diagnosed as UCAN, and all were classified as LGD (Figure 8). Two of the three patients presented with synchronous multiple lesions that were clearly delineated by CV‐SCAN. Of the 22 biopsies taken from CV‐N–positive areas, 20 were histologically confirmed as dysplasia. Among the nine biopsies from adjacent CV‐N–negative areas, eight were confirmed to be non‐dysplastic (Table 5).

**TABLE 5:** Characteristics of biopsy specimens diagnosed with ulcerative colitis‐associated neoplasia (UCAN) by CV‐SCAN (crystal violet staining for colitis‐associated neoplasia).

		Pathological diagnosis		Total
		UCAN	Non‐UCAN	
CV‐N	Positive	20	2	22
	Negative	1	8	9
Total		21	10	31

*Note:* A total of 31 biopsy specimens—including both neoplastic and non‐neoplastic samples—were obtained from 3 patients with 8 lesions diagnosed as UCAN based on crystal violet staining. Of these, 22 specimens exhibited Crystal Violet–Neoplasia (CV‐N) staining. Among the CV‐N–positive specimens, 20 were histopathologically confirmed as UCAN. Based on these results, the sensitivity and specificity of the CV‐N pattern for detecting UCAN were 95.2% and 80.0%, respectively.

Six of the eight lesions were not detectable under white light endoscopy but became visible after CV‐SCAN and were subsequently diagnosed as UCAN. The remaining two appeared as indistinct reddish areas, in which CV‐SCAN clarified lesion extent.

### 3.4 Comparison of mRNA Expression Profiles in CV‐P Stained vs. Adjacent Non‐Stained Areas

Biopsy specimens for microarray analysis were obtained from two individuals. As controls, samples of normal colorectal mucosa were collected from a patient without UC undergoing examination for colorectal adenoma. In one patient with UC, three biopsy specimens were taken from an area with intense CV‐P staining and three from adjacent non‐stained mucosa. Thus, a total of three samples were obtained for each condition: control, stained, and non‐stained.

**FIGURE 9:**
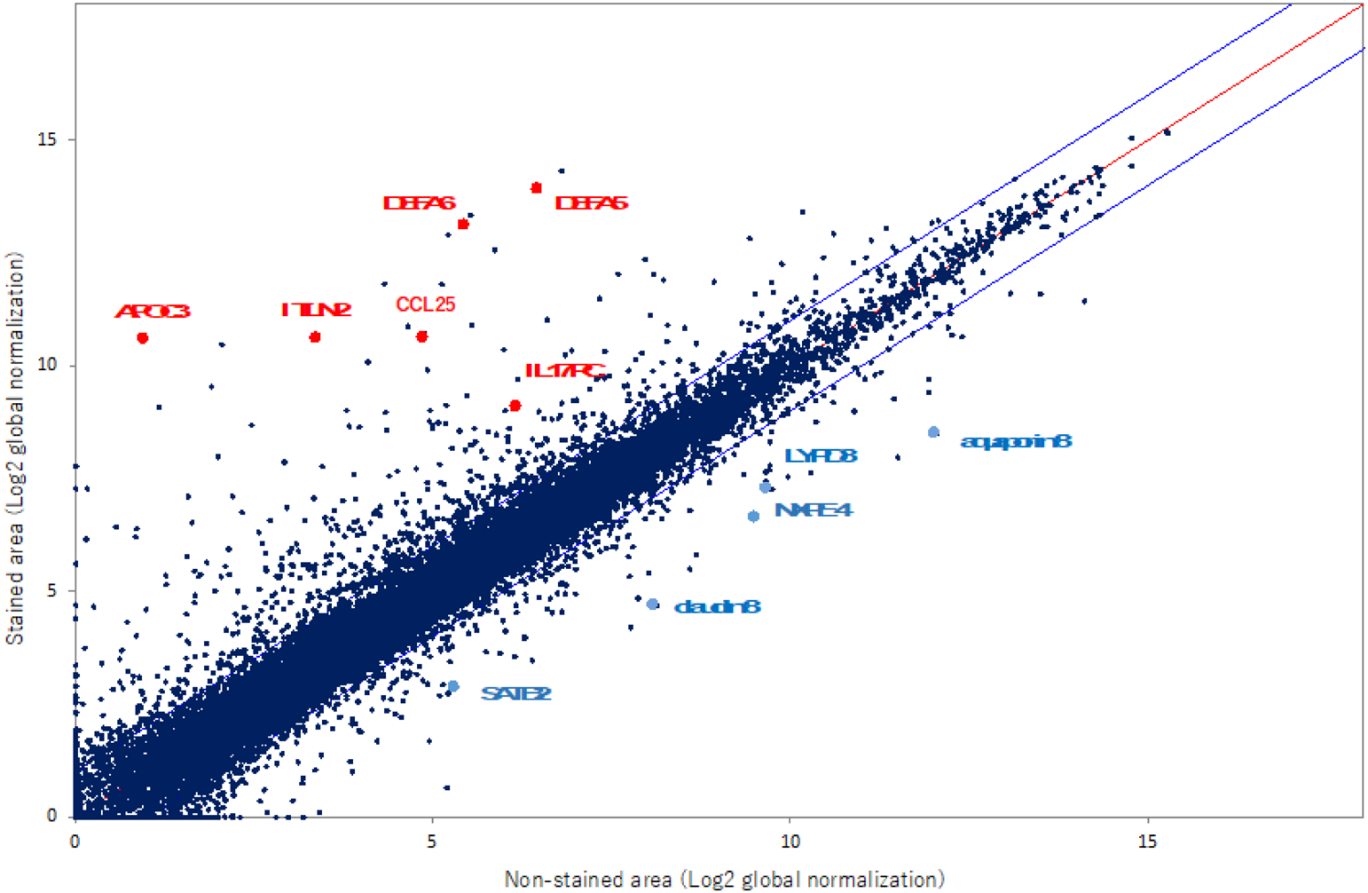
Comparison of mRNA expression profiles between stained and non‐stained areas using microarray analysis. Each value in the scatter plot represents the log_2_‐transformed expression intensity, normalized to a median signal value of 25. The x‐axis indicates mRNA expression levels in non‐stained areas, and the y‐axis indicates levels in stained areas. In stained areas, mRNAs specifically associated with Paneth cells—including defensin α‐5 (DEFA5), defensin α‐6 (DEFA6), and intelectin 2 (ITLN2)—were markedly upregulated, indicating a Paneth cell metaplasia phenotype. Conversely, expression of neurexophilin and PC‐esterase domain family member 4 (NXPE4) was downregulated in these areas. Additionally, genes typically expressed in the small intestine, such as apolipoprotein C‐III (APOC3) and C‐C motif chemokine ligand 25 (CCL25), were also upregulated. In contrast, several genes associated with colonic epithelial integrity and function were downregulated, including claudin 8 (CLDN8), which is involved in tight junctions; aquaporin 8 (AQP8), a water channel protein; and Ly6/PLAUR domain‐containing 8 (LYPD8), which plays a role in mucosal defense by inhibiting bacterial adhesion in the colon. Furthermore, special AT‐rich sequence‐binding protein 2 (SATB2)—a transcription factor specifically expressed in colonic epithelial cells and known to suppress small intestinal differentiation—was significantly decreased. This suggests a shift from colonic toward small intestinal epithelial characteristics in stained areas.

Microarray analysis of CV‐P–stained areas showed increased expression of Paneth cell–specific genes (DEFA5, DEFA6, ITLN2), UCAN‐associated IL17RC, and small intestine–related genes (APOC3, CCL25), together with reduced expression of NXPE4, barrier‐related genes (claudin 8, aquaporin 8, LYPD8), and the colon‐specific stem cell marker SATB2 (Figure 9).

**FIGURE 10:**
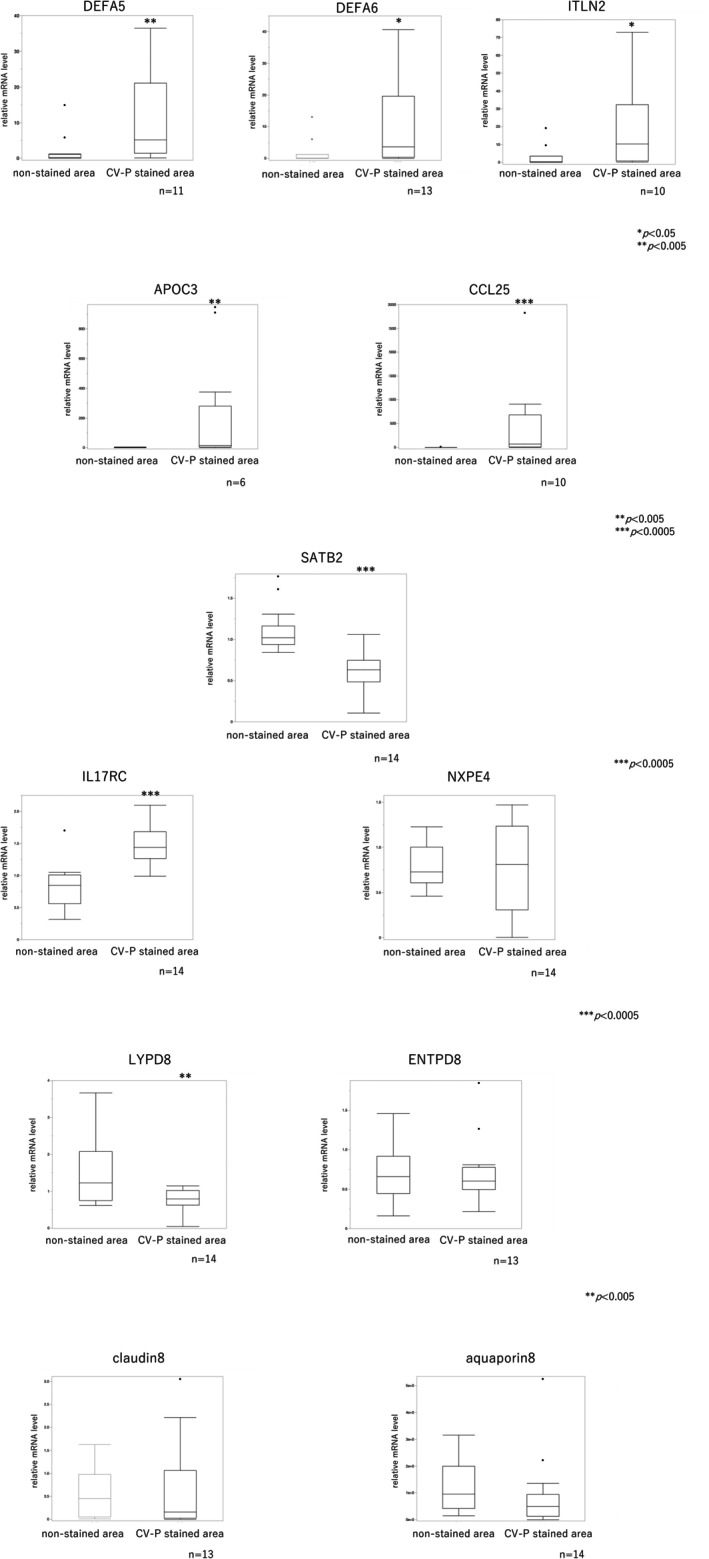
Differential mRNA expression in stained versus non‐stained areas as assessed by quantitative PCR. Quantitative PCR analysis revealed significantly higher expression levels of defensin α‐5 (DEFA5), defensin α‐6 (DEFA6), and intelectin 2 (ITLN2)—genes specific to Paneth cells—in stained areas. Interleukin 17 receptor C (IL17RC) was also significantly upregulated in these areas. Additionally, apolipoprotein C‐III (APOC3) and C‐C motif chemokine ligand 25 (CCL25), both typically expressed in the small intestine, showed significantly higher expression. In contrast, neurexophilin and PC‐esterase domain family member 4 (NXPE4), claudin 8 (CLDN8), and aquaporin 8 (AQP8) did not show significant differences between stained and non‐stained areas. Ly6/PLAUR domain‐containing 8 (LYPD8), a gene specifically expressed in the colonic epithelium and involved in antimicrobial defense, was significantly downregulated in stained areas. Similarly, special AT‐rich sequence‐binding protein 2 (SATB2), a key transcription factor for maintaining colonic epithelial identity, was also significantly decreased. No significant difference was observed in ectonucleoside triphosphate diphosphohydrolase 8 (ENTPD8) expression, which is known to degrade ATP released from intestinal bacteria and is implicated in preventing severe colitis (*p < 0.05, **p < 0.005, ***p < 0.0005).

Subsequently, we compared mRNA expression between CV‐P–stained and adjacent non‐stained areas in the same patients using quantitative reverse transcription PCR (qRT‐PCR) in 14 patients (Table 6). This analysis confirmed significantly increased expression of DEFA5, DEFA6, ITLN2, IL17RC, APOC3, and CCL25 in stained areas. In contrast, LYPD8 and SATB2 expression levels were significantly decreased in stained areas. No statistically significant differences were observed in the expression of NXPE4, claudin 8, aquaporin 8, or ENTPD8 (Figure 10).

**TABLE 6:** Cases subjected to quantitative RT‐PCR analysis.

Sex (male/female)	9/5
Age	25–81 years old (median, 56.5 years old)
Disease extent	Pancolitis: 12, left‐sided colitis: 2
Disease duration	4–22 years (median, 10.5 years)
History of UCAN	0

*Note:* mRNA expression was analyzed by quantitative reverse transcription PCR (RT‐PCR) in 14 patients with ulcerative colitis (UC). All patients had biopsy specimens from areas of crystal violet staining suspected to contain Paneth cell metaplasia (PCM), as well as from adjacent non‐stained areas.

## 4 Discussion

We developed a novel endoscopic staining technique, CV‐SCAN, using ultra‐diluted CV. We evaluated the diagnostic accuracy of CV‐SCAN for detecting PCM and UCAN and confirmed its high accuracy for identifying PCM. Moreover, it may enhance the detection rate of previously undetectable PCM and UCAN lesions.

The endoscopic detection of PCM may provide an additional dimension for assessing chronic inflammatory burden and cancer risk in UC. Previous histopathological studies have shown that the distribution of PCM correlates with the duration and extent of chronic inflammation [11]. In our cohort, CV‐SCAN–positive areas were more frequent in patients with longer disease duration and were significantly associated with a history of UCAN. CV‐SCAN–positive staining during remission may therefore reflect cumulative inflammatory remodeling, even when conventional endoscopic findings are unremarkable.

We also observed that staining patterns provided useful information for differentiating PCM from UCAN. PCM typically appeared as uniformly dark purple areas with tubular pits and smaller lesion sizes, whereas UCAN lesions showed heterogeneous, pale purple staining with densely packed small round pits and tended to be larger.

Comparative pathological and molecular analyses of stained and non‐stained areas revealed upregulation of Paneth cell–specific genes (DEFA5, DEFA6, ITLN2) and small intestine–specific genes (CCL25, APOC3) in the stained areas. These findings suggest a molecular phenotype resembling small intestinal metaplasia, which is often difficult to distinguish histologically. Additionally, expression of IL17RC—a gene implicated in the pathogenesis of UCAN—was increased, while SATB2, a colon‐specific stem cell marker, was consistently downregulated in stained areas [13, 14, 15, 18, 19, 20]. These findings are consistent with reports linking SATB2 loss and miR‐31 upregulation to UCAN and small intestinal type metaplasia [13, 14, 15, 21], and together suggest that CV‐SCAN–positive areas may represent a precancerous state. Interestingly, molecular expression profiles differed markedly between CV‐SCAN–positive and –negative areas. This suggests that CV‐SCAN may discriminate lesions that are molecularly distinct but not visible using conventional endoscopic techniques. It is also possible that small intestinal metaplasia in UC does not occur diffusely throughout the colonic mucosa, but instead arises in specific areas and gradually spreads to form clearly demarcated areas.

Several CV‐SCAN–positive areas were negative for PCM on histology. This discrepancy may be due partly to the low sensitivity of PCM as a marker of small intestinal metaplasia and partly to sampling limitations, because very small stained areas were biopsied and the exact microscopic region containing Paneth cells may not have been captured. Longitudinal studies are needed to clarify whether CV‐SCAN–positive/PCM‐negative regions subsequently develop PCM or UCAN.

The mechanisms underlying the appearance of CV‐SCAN–positive areas are also of interest. CV is a cationic dye that binds to nucleic acids and other negatively charged cellular components, which are abundant in highly proliferative or secretory cells such as Paneth cells and dysplastic epithelium. Chronic inflammation in UC promotes crypt distortion, nuclear crowding, and increased nucleic acid synthesis, potentially enhancing dye uptake. In addition, differences in mucus composition and thickness between colonic and small intestinal type mucosa may alter dye penetration [22, 23].

Safety considerations are crucial for the clinical application of CV‐SCAN. Although CV has been widely used in chromoendoscopy, animal studies have reported carcinogenicity with high‐dose, long‐term oral exposure [24]. In our protocol, an ultra‐diluted 0.006% solution was applied in a small volume (~40 mL) and limited to the left colon. No adverse events were observed, but cumulative exposure over repeated procedures remains a concern. Limiting CV use solely by disease duration, however, may not be appropriate. A practical strategy may be to perform CV‐SCAN once in each patient and, if negative, extend the interval before repeating the procedure, thereby minimizing cumulative exposure while preserving diagnostic benefit. In line with current recommendations [25], CV‐SCAN should be restricted to selected high‐risk patients, used in minimal quantities, and performed under appropriate ethical oversight. The development of alternative, safer staining agents remains an important future goal.

This study has several limitations. It was a single‐center retrospective study with a limited number of UCAN lesions (eight lesions in three patients), which restricts generalizability and statistical power. CV‐SCAN was applied only in the left colon, and its performance in the right colon was not evaluated. Molecular analyses were based on a relatively small number of biopsy specimens and focused mainly on CV‐P–positive areas. Corresponding white light images were not consistently available for all CV‐SCAN–positive sites, limiting our ability to provide paired imaging examples for every lesion. Finally, staining assessment included some subjective elements, although the clear distinction between CV‐P and CV‐N patterns suggests that standardized criteria could be established.

In summary, CV‐SCAN is a novel endoscopic staining technique that enables visualization of PCM and early dysplastic changes in UC. CV‐SCAN–positive areas exhibit molecular features of small intestinal type metaplasia and are associated with a history of UCAN, suggesting that they may represent a precancerous mucosal field. The CV‐N pattern is highly sensitive for detecting LGD, including lesions that are inconspicuous on white light endoscopy. CV‐SCAN may thus enhance risk stratification and guide targeted surveillance and treatment strategies in patients with UC. Further multicenter prospective studies with long‐term follow‐up are warranted to validate these findings and to clarify the optimal indications and safety profile of CV‐SCAN in clinical practice.

## Author Contributions

A.T. designed the study, drafted the manuscript and collected and analyzed the data. N.C., C.K., Y.O., K.N., M.H., S.K., and R.H. supervised the study and critically revised the manuscript. All authors have read and approved the final manuscript and agree to be accountable for all aspects of the work.

## Funding

This work was supported by National Defense Medical College.

## Ethics Statement

This study was conducted in accordance with the Declaration of Helsinki and was approved by the institutional review board of National Defense Medical College Hospital (Approval No. 4957). Written informed consent was obtained from all participants prior to their inclusion in the study.

## Conflicts of Interest

The authors declare no conflicts of interest.
